# Oleanolic Acid Enhances the Beneficial Effects of Preconditioning on PC12 Cells

**DOI:** 10.1155/2014/929854

**Published:** 2014-11-13

**Authors:** Babongile C. Ndlovu, Willie M. U. Daniels, Musa V. Mabandla

**Affiliations:** Department of Human Physiology, School of Laboratory Medicine and Medical Sciences, College of Health Sciences, University of KwaZulu-Natal, Westville Campus, Durban 4000, South Africa

## Abstract

Preconditioning triggers endogenous protection against subsequent exposure to higher concentrations of a neurotoxin. In this study, we investigated whether exposure to oleanolic acid (OA) enhances the protective effects of preconditioning on PC12 cells exposed to 6-hydroxydopamine (6-OHDA). A concentration response curve was constructed using 6-OHDA (50, 150, 300, and 600 *μ*M). The experiment consisted of 6 groups: untreated, OA only, Group 1: cells treated with 6-OHDA (50 *μ*M) for 1 hour, Group 2: cells treated with 6-OHDA (150 *μ*M) for 1 hour, Group 3: cells treated with 6-OHDA (50 *μ*M) for 30 minutes followed 6 hours later by treatment with 6-OHDA (150 *μ*M) for 30 minutes, and Group 4: cells treated as in group 3 but also received OA immediately after the second 6-OHDA treatment. Cell viability and apoptotic ratio were assessed using the MTT and Annexin V staining tests, respectively. In preconditioned cells, we found that cell viability remained high following exposure to 6-OHDA (150 *μ*M). OA treatment enhanced the protective effects of preconditioning. Similarly, with the annexin V apoptosis test, preconditioning protected the cell and this was enhanced by OA. Therefore, preexposure of PC12 cells to low 6-OHDA concentration can protect against subsequent toxic insults of 6-OHDA and OA enhances this protection.

## 1. Introduction

The formation of free radicals and an impaired ability of cells to resist stress can result in damage to all major macromolecules in cells including proteins, nucleic acids, and lipids [1]. Living cells have a way of resisting oxidative stress; these include detoxification, production of protein chaperones such as heat-shock proteins, removal of damaged molecules, and increase of the levels of DNA repair enzymes [1]. The latter enzymes are enhanced when cells are exposed to mild stress and this is called preconditioning/hormesis [1–3]. Preconditioning is therefore a process by which cells are exposed to mild oxidative stress so as to make them more resistant when subsequently exposed to more severe oxidative stress [1]. During preconditioning bioprotective mechanisms that include induction of cytoprotective pathways (molecular chaperones), antioxidative systems, DNA repair systems, and immune system are stimulated. These molecular mechanisms have been shown to protect cells from various forms of cell death [4, 5].

Parkinson's disease is a neurodegenerative disease and its prevalence rate is continuing to escalate as the life expectancy of the general population increases. Despite numerous studies its management remains unsatisfactory. Cell based therapy is one of the emerging treatments of Parkinson's disease and its use has grown in promise since certain stem cells have also been shown to have the ability to enhance endogenous neurogenesis [5]. However, the harsh environment of the diseased brain is a severe threat to the survival and/or correct differentiation of these implanted cells [5].

It has been shown that preconditioning may also occur within cells of the nervous system [6]. This study demonstrated how exposure of a dopamine cell line to sublethal concentrations of 6-hydroxydopamine (6-OHDA) protected against subsequent exposure to high concentrations of 6-OHDA. These results suggested that dopaminergic cells, when treated with a low concentration of a chemical, may enable the cells to survive better in the neurodegenerative brain.

Oleanolic acid (OA) is a relatively nontoxic compound that has been shown to have antitumoric [7], hepatoprotective [8], anti-inflammatory [9], antihyperlipidemic [8], antihyperglycemic or hypoglycaemic [10], and antimicrobial [8] properties, in addition to being an analgesic, antiulcer, anti-infertility, and anticarcinogenic agent [7]. Oleanolic acid therefore seems to have a variety of health-promoting/disease-preventing characteristics. In this study we investigated the effects of preconditioning on cell viability of PC12 cells and assessed whether OA treatment would enhance the protective effects of preconditioning.

## 2. Materials and Methods

### 2.1. Chemicals and Reagents

We obtained the adrenal phaeochromocytoma cell line (generally referred to as PC12 cell line) from the Department of Biochemistry, University of KwaZulu-Natal (South Africa). RPMI-1640 growth medium was purchased from Highveld Biological (PTY) Ltd (South Africa). Heat-inactivated horse serum, fetal calf serum, 6-OHDA, trypsin, dimethyl thiazolyl diphenyltetrazolium salt (MTT), and OA were purchased from Sigma-Aldrich (South Africa). Ascorbic acid was obtained from SAARchem (South Africa). Penicillin/streptomycin solution was obtained from Biochrom AG (South Africa). The Annexin V kit was purchased from BD Biosciences (South Africa). All solutions were prepared fresh prior to each experiment or assay.

### 2.2. Cell Culture

The PC12 cells were grown in tissue culture corning flasks (Whitehead Scientific, South Africa). RPMI-1640 medium containing 300 mg/L L-glutamine, 4.5 g/L D-glucose, 1.5 g/L sodium bicarbonate, 1 mM sodium pyruvate, and 10 mM HEPES buffer was used as a component of growth medium in the following manner: growth medium which was made up of 83% RPMI-1640, 10% heat-inactivated horse serum, 5% fetal calf serum, and 2% penicillin-streptomycin solution (penicillin 10.000 U/mL, streptomycin 10.000 *µ*g/mL) and was kept at 37°C in a humidified incubator supplied with 5% carbon dioxide (CO_2_). Cells were grown until 70–80% confluency was reached. When enough cells were grown, cells were then trypsinised, a cell count was performed using a Neubauer counting chamber under light microscope and the cells were then plated in a 96-well microtitre plate (for the MTT assay) and a 24-well plate (for the annexin V apoptosis test), at plating densities of 50 000 and 1000 000 cells per well, respectively. Cells were left to adhere to the plate surfaces overnight (12 up to 15 hours). On the day of cell treatment the medium in the wells was aspirated and cells were treated with 6-OHDA dissolved in medium or medium alone. 6-OHDA stock solution was prepared using a saline solution containing 2% ascorbic acid. All solutions were diluted with serum-free medium to their final concentration. After treatment, media with toxin was aspirated and cells were incubated with serum-free media for 24 hours before cell viability and apoptosis assays were conducted.

### 2.3. Concentration-Response Curve

Four concentrations of 6-OHDA (50, 150, 300, and 600 *μ*M) were used to treat cells for a duration of 1 hour. Twenty-four hours later the MTT assay and Annexin V staining tests were performed to assess cell viability and apoptotic ratio, respectively.

### 2.4. Preconditioning Regimen

Six groups of cells were used in the experiment. These consisted of 2 control groups (untreated cells and cells treated with OA (5 *µ*M) only). Group 1 cells (50 *µ*M) and Group 2 cells (150 *µ*M) were exposed to 6-OHDA for 1 hour. Group 3 cells were exposed to 6-OHDA (50 *µ*M) for 30 minutes before being exposed 6 hours later to 6-OHDA (150 *µ*M) for a further 30 minutes. Group 4 cells were treated as in Group 3 but were also treated with OA (5 *µ*M) for a period of 24 hours after the completion of the protocol used for Group 3 cells. The viability of cells and apoptotic ratio were assessed 24 hours later by the MTT viability test followed by the Annexin V apoptosis test.

### 2.5. Cell Viability Testing: MTT Procedure

The MTT assay was used to test for the viability of PC12 cells. The cells were plated at a density of 50 thousand cells per well in a 96-well microtiter plate 12 to 15 hours before experimentation. After each cell treatment protocol mentioned in the previous section, the cells were incubated in a 37°C incubator for 24 hours. Following this, the cells were incubated with 20 *µ*L of MTT (5 mg/mL) for 3.5 hours at 37°C in an incubator. After the incubation, media was removed without disturbing the cells and DMSO (150 *µ*L) was added to dissolve the formazan crystals. The plates were shielded from light using a foil and left in an orbital shaker maintained at 600 revolutions/minute for 15 minutes. The resulting purple solution was measured spectrophotometrically. Cells with normal functioning mitochondria that are metabolically active and proliferating produce an increase in the amount of MTT formazan formed and hence an increase in absorbance. The amount of MTT formazan product formed was determined by measuring absorbance (A) using a microplate reader (Bio-Tek) at a wavelength of 630 nm.

### 2.6. Annexin V-Propidium Iodide Procedure

The Annexin V assay was used to measure the apoptotic ratio (portion of apoptotic cells: portion of viable cells) of PC12 cells. The cells were plated at a density of 1 million cells per well in a 24-well plate 12 to 15 hours before experimentation. During the cell treatment protocol, cells were incubated with serum-free medium. Following this, the cells were processed as follows. The media was discarded from all the wells and cells were washed twice in 0.90% w/v phosphate buffered saline (PBS, 500 *µ*L). Trypsin was added to each well (just enough to cover the surface) and incubation occurred for 5 minutes in a 35°C heated incubator. Following incubation, the plates were lightly tapped until all the cells were detached from the well surface. PBS (500 *µ*L) was added to each well to neutralize the trypsin. The cell suspension from each well was transferred to a Falcon tube, pipetted up and down to break up the clumps, and centrifuged at 12,000 g in a refrigerated centrifuge (Hermle Labortechnik GmbH, Germany) for 5 minutes at 4°C after which the supernatant was removed, leaving the pellet in the tube.

### 2.7. Staining Procedure

Binding buffer was prepared according to the manufacturer's instructions (BD Pharminogen) and 100 *µ*L was added to the cell suspension into the Falcon tube. FITC Annexin V (5 *µ*L) was added to each sample followed by the addition of propidium iodide (5 *µ*L). The tube(s) were mixed on a vortex mixer, incubated for 20 minutes at room temperature (25°C) in the dark after which binding buffer (400 *µ*L) was added to each tube. The reaction tube was analyzed by flow cytometry (BD FacsCalibur) within 1 hour (each sample was mixed on a vortex mixer before reading).

### 2.8. Statistical Analysis

The data were analyzed using the software program GraphPad Prism (version 5). Results are reported as mean ± SEM of 3 experiments. Data were subjected to Shapiro-Wilk normality testing and found to have a skewed distribution. Subsequently nonparametric tests were performed (Kruskal-Wallis test followed by Mann-Whitney *U* test). A *P* value less than 0.05 was considered significant.

## 3. Results

### 3.1. Concentration-Response Curve

**Figure 1:**
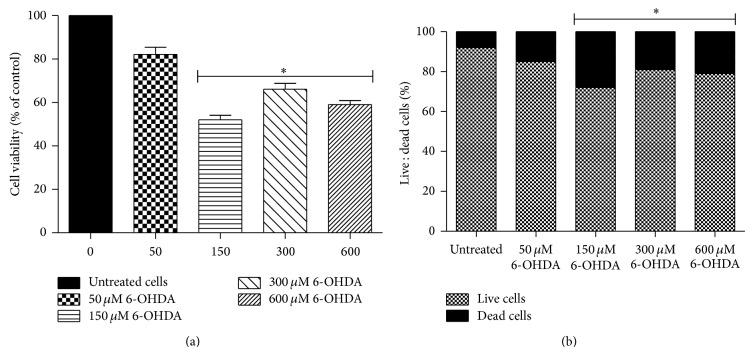
Effects of 6-OHDA on (a) cell viability of PC12 cells as shown by the MTT assay and (b) apoptotic ratio of PC12 cells as shown by the Annexin V apoptosis test 24 hours after cell treatment. Four concentrations of 6-OHDA were used: 50 *μ*M, 150 *μ*M, 300 *μ*M, and 600 *μ*M. ^*^0 *μ*M versus 150, 300, and 600 *μ*M, *P* < 0.05.

There was a neurotoxin effect on PC12 cells treated with 150 *µ*M, 300 *µ*M, and 600 *µ*M 6-OHDA) ^*^(0 *µ*M versus 150, 300, and 600 *µ*M, *P* < 0.05, Figures 1(a) and 1(b)). There was minimal cell damage in the group treated with the 50 *µ*M concentration of 6-OHDA (Figures 1(a) and 1(b)).

### 3.2. Preconditioning and OA Treatment following Exposure to 6-OHDA

**Figure 2:**
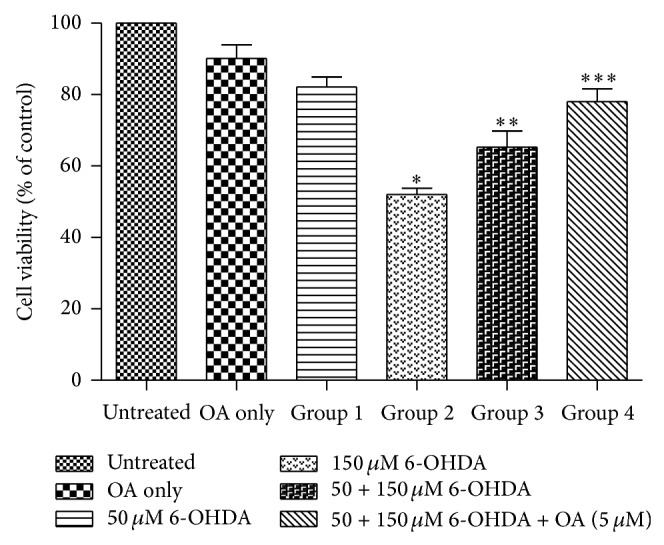
Graph showing the synergistic effect of OA and 6-OHDA on preconditioned PC12 cells assessed by the MTT assay. ^*^(Untreated group versus Group 2, *P* < 0.05), ^**^(Group 2 versus Group 3, *P* < 0.05), and ^***^(Group 3 versus Group 4, *P* < 0.05).

There was a neurotoxin effect on cells exposed to a higher concentration of 6-OHDA (150 *µ*M) ^*^(Untreated group versus Group 2, *P* < 0.05, Figure 2). There was a neuroprotective effect on cells initially exposed to a lower concentration of 6-OHDA (50 *µ*M) ^**^(Group 2 versus Group 3, *P* < 0.05, Figure 2). This neuroprotection was further enhanced by exposure to OA ^***^(Group 3 versus Group 4).

**Figure 3:**
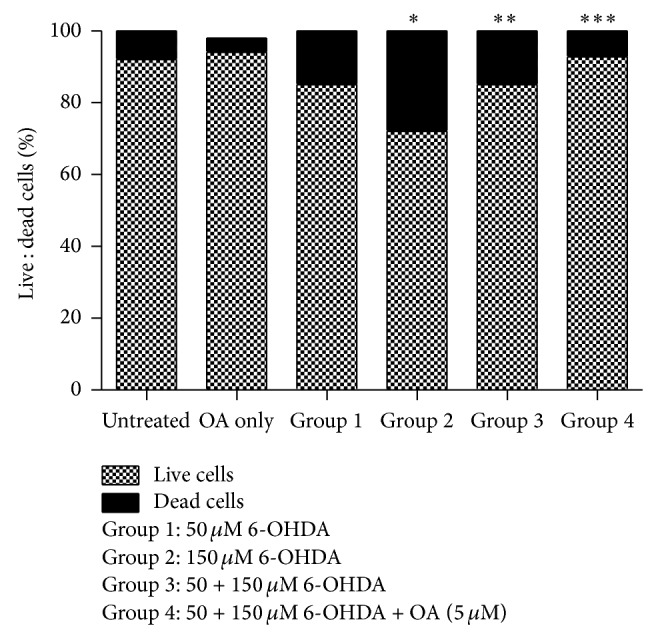
Graph showing the synergistic effect of OA and 6-OHDA on preconditioned PC12 cells assessed by the Annexin V assay of apoptotic cells. ^*^(Untreated group versus Group 2, *P* < 0.05), ^**^(Group 2 versus Group 3, *P* < 0.05), and ^***^(Group 3 versus Group 4).

Similarly when using the Annexin V assay to ascertain apoptosis we found that there was a neurotoxic effect on cells exposed to a higher concentration of 6-OHDA (150 *µ*M) ^*^(Untreated group versus Group 2, *P* < 0.05, Figure 3). There was a neuroprotective effect on cells initially exposed to a lower concentration of 6-OHDA (50 *µ*M) ^**^(Group 2 versus Groups 3, *P* < 0.05, Figure 2). This neuroprotection was further enhanced by exposure to OA ^***^(Group 3 versus Group 4).

## 4. Discussion

6-OHDA has been shown to be an effective neurotoxin to study the pathogenesis of Parkinson's disease both in vitro and in vivo [11, 12]. Previous studies have shown that 6-OHDA inhibits mitochondrial respiration, generates intracellular reactive oxygen species, induces abnormal cell cycle reentry, and eventually causes dopaminergic neuron death [11]. However, sublethal levels of 6-OHDA have been shown to provide cellular protection against a subsequent toxic concentration of 6-OHDA in a phenomenon called preconditioning/hormesis [6]. In this study we investigated the combined effect of preconditioning and oleanolic acid (OA) on cultured PC12 cells.

In the study we found that treating the cells with 150 *μ*M 6-OHDA induced oxidative stress by initiating a sequence of events that reduce cellular activity leading to apoptotic cell death. We showed that preconditioning PC12 cells by exposing them to a lower concentration of 6-OHDA for a short period before subsequent exposure to a higher concentration resulted in greater cell viability and therefore less cell death. This is similar to a study that showed that the use of a sublethal concentration of 6-OHDA prior to exposure to a toxic concentration protected the cells by activating the antioxidant response system [6]. In our study we showed that this neuroprotective effect of preconditioning was enhanced by subsequent exposure of the cells to the triterpenoid oleanolic acid.

Differences in the intensity, duration, and/or frequency of a particular stress stimulus determines whether that stimulus is strong enough to elicit a response of sufficient magnitude to serve as a preconditioning trigger, or whether it is too robust and therefore harmful [13]. In comparable experiments to our study, it was shown that the administration of small and adaptive concentrations of H_2_O_2_ to PC12 cells protected these cells against subsequent oxidative damage induced by paraquat and 6-OHDA [14]. The protective function of H_2_O_2_ was attributed to the activation of antioxidative enzymes in the cells [14]. It has also been suggested that protection by preconditioning may involve the MAPK and PI3K/Akt pathways [15]. These signalling pathways are important in regulating apoptotic cell death in development and in disease states [15]. These results therefore suggest that preconditioning may be a promising approach to treating reactive oxygen species-mediated diseases such as Parkinson's disease [14].

Although the mechanism by which oleanolic acid provides protection against oxidative stress is unknown, most studies that look at synthetic analogues of oleanolic acid have shown that it activates the Nrf2/ARE signalling pathway which controls the upregulation of an array of genes involved in antioxidant responses, heat shock chaperone proteins, and mitochondrial protective genes [16–18]. Oleanolic acid treatment has been shown to result in the retention of higher glutathione levels in oxidatively stressed cells [19]. Oleanolic acid treatment also restores the levels of glutathione peroxidase, catalase, and superoxide dismutase in oxidatively stressed PC12 cells [19]. It has been shown that oleanolic acid decreases the formation of malondialdehyde which is a marker for the presence of oxidative stress [19]. It has also been shown that the concentrations of interleukin-6 (IL-6) and tumour necrosis factor alpha (TNF-*α*) increase in oxidatively stressed PC12 cells; however, this increase is attenuated by exposure to oleanolic acid [19]. IL-6 and TNF-*α* have also been shown to play a role in the neuroinflammation associated with Parkinson's disease [20]. The neuroprotective effects of oleanolic acid have also been investigated in an animal model for multiple sclerosis suggesting a role for oleanolic acid in other neuroinflammatory diseases [21]. In our study we were able to show that the addition of 5 *µ*M oleanolic acid [22] exacerbated the neuroprotective effects of preconditioned dopamine containing cell lines.

Although a majority of studies have looked at the effects of modified and biologically enhanced molecules, in our study we used a parent molecule, oleanolic acid, and our results showed the beneficial effects of this molecule in a cell culture model of oxidative stress.

## 5. Conclusion

Exposing stem cells to preconditioning in stem cell based therapy can make these cells more resistant to the toxic environment present in neurodegenerative brains such as in Parkinson's disease. This may be enhanced by treating these stem cells with oleanolic acid which may attenuate or slow down the disease process, therefore sustaining the improved quality of life in Parkinson's disease patients undergoing this treatment.
